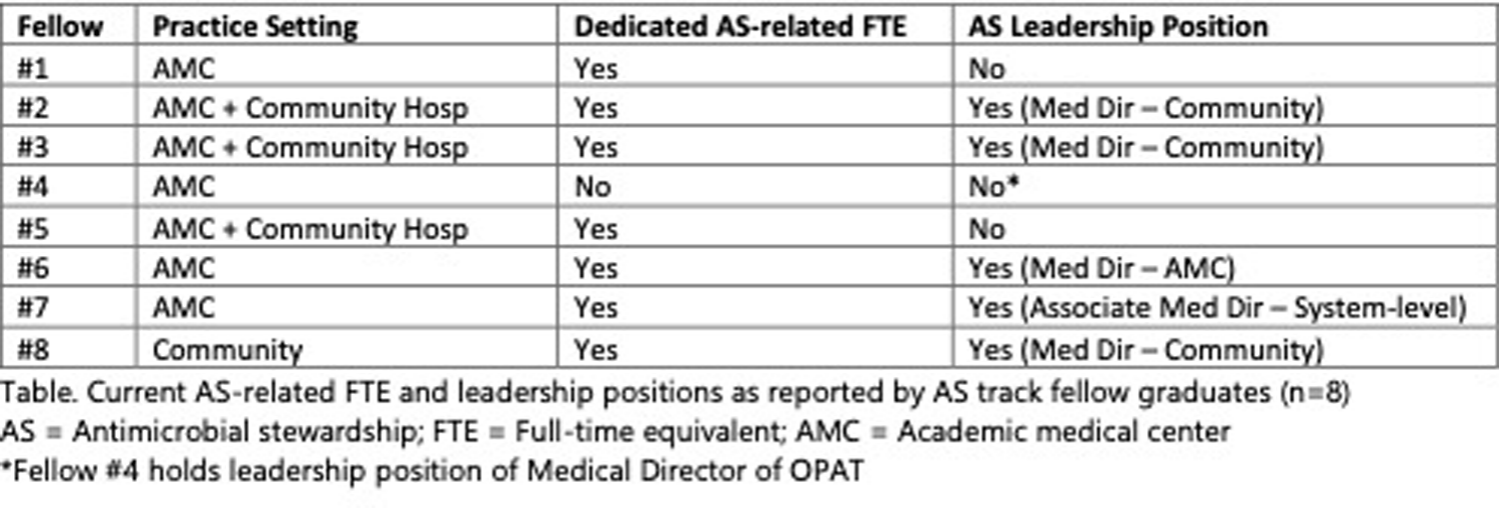# 108 CLABSI designation following hepato-pancreato-biliary surgery

**DOI:** 10.1017/ash.2026.10525

**Published:** 2026-06-23

**Authors:** Corina Lopez, Howard Gold, Preeti Mehrotra, Wendy Stead, Sharon Wright, Matthew Lee

**Affiliations:** 1 Beth Israel Deaconess Medical Center; 2 Bidmc; 3 Beth Israel Lahey Health

## Abstract

**Background:** Many Infectious Diseases (ID) fellowship programs have created antimicrobial stewardship (AS) scholarly tracks to guide burgeoning stewards. However, few programs have reported details of their AS curricula or career outcomes after track completion. We describe the major components of our AS track and the current AS-related faculty roles of prior participants. **Methods:** In 2015, our ID fellowship program, based at an academic medical center (AMC), formally created an AS track for second-year fellows (typically one fellow per year). The goal is to prepare fellows to become effective AS team members and potentially hold program leadership roles. We annually survey prior track participants regarding their current institution and roles. All current fellows are surveyed annually to evaluate AS-related rotations and conference series using a five-point Likert scale (1=Poor to 5=Excellent). **Results:** The track emphasizes three major components (Education, Mentorship, and AS Experiences) based on the SHEA White Paper “Guidance for the Knowledge and Skills Required for Antimicrobial Stewardship Leaders” (Cosgrove SE, ICHE 2014) (Figure 1). Early stewardship content education is provided through an annual conference series (“Summer Series”) and an experiential combined AS/Infection Control rotation for first-year fellows; both have demonstrated improved evaluation scores over the past five years (Figure 2). Since 2015, eight fellows have completed the second-year AS track; the majority currently practice at AMCs. Seven (88%; 7/8) currently report dedicated FTE support for AS activities and five (63%; 5/8) hold AS leadership positions as either Medical Director or Associate Medical Director (Table). **Conclusion:** During the first decade of our AS scholarly track, we developed a structured curriculum emphasizing acquisition of stewardship expertise, mentorship, and diverse experiential training. The majority of AS track fellow graduates continue in stewardship roles as faculty, including leadership positions, suggesting our longitudinal track may support early-career development in AS.

**Figure:**
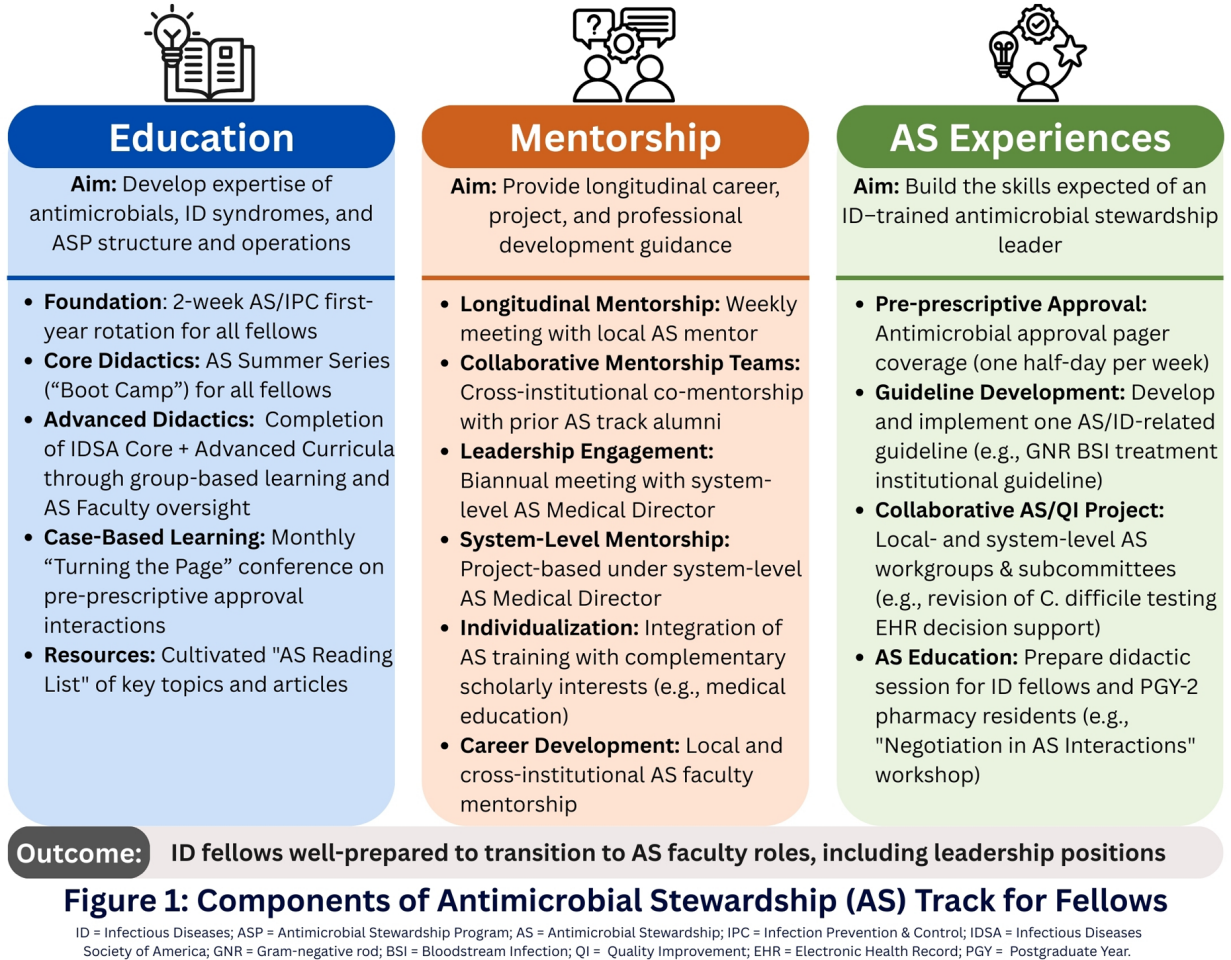

**Figure:**
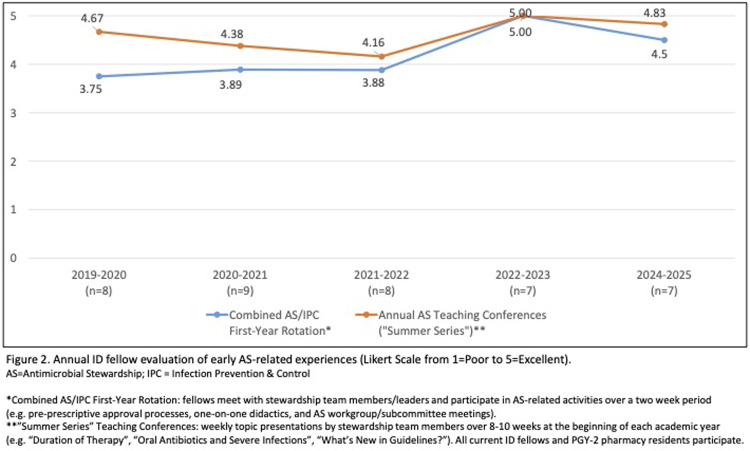

**Figure:**